# Factors affecting 30-day mortality in poor-grade aneurysmal subarachnoid hemorrhage: a 10-year single-center experience

**DOI:** 10.3389/fneur.2024.1286862

**Published:** 2024-02-15

**Authors:** Antonino Scibilia, Arianna Rustici, Marta Linari, Corrado Zenesini, Laura Maria Beatrice Belotti, Massimo Dall’Olio, Ciro Princiotta, Andrea Cuoci, Raffaele Aspide, Ernesto Migliorino, Manuel Moneti, Carmelo Sturiale, Carlo Alberto Castioni, Alfredo Conti, Carlo Bortolotti, Luigi Cirillo

**Affiliations:** ^1^IRCCS Istituto delle Scienze Neurologiche di Bologna, UOC Neurochirurgia, Bologna, Italy; ^2^IRCCS Istituto delle Scienze Neurologiche di Bologna, UOSI di Neuroradiologia Ospedale Maggiore, Bologna, Italy; ^3^Dipartimento di Scienze Biomediche e Neuromotorie (DIBINEM), Università di Bologna, Bologna, Italy; ^4^IRCCS Istituto delle Scienze Neurologiche di Bologna, Unità di Epidemiologia e Biostatistica, Bologna, Italy; ^5^IRCCS Istituto delle Scienze Neurologiche di Bologna, UOC Neuroradiologia, Bologna, Italy; ^6^IRCCS Istituto delle Scienze Neurologiche di Bologna, UOC Anestesia e Rianimazione, Bologna, Italy

**Keywords:** poor-grade, subarachnoid hemorrhage, predictors, Mortality, intracranial aneurysms

## Abstract

**Background:**

The management of patients with poor-grade aneurysmal subarachnoid hemorrhage (aSAH) is burdened by an unfavorable prognosis even with aggressive treatment. The aim of the present study is to investigate the risk factors affecting 30-day mortality in poor-grade aSAH patients.

**Methods:**

We performed a retrospective analysis of a prospectively collected database of poor-grade aSAH patients (World Federation of Neurosurgical Societies, WFNS, grades IV and V) treated at our institution from December 2010 to December 2020. For all variables, percentages of frequency distributions were analyzed. Contingency tables (Chi-squared test) were used to assess the association between categorical variables and outcomes in the univariable analysis. Multivariable analysis was performed by using the multiple logistic regression method to estimate the odds ratio (OR) for 30-day mortality.

**Results:**

A total of 149 patients were included of which 32% had WFNS grade 4 and 68% had WFNS grade 5. The overall 1-month mortality rate was 21%. On univariable analysis, five variables were found to be associated with the likelihood of death, including intraventricular hemorrhage (IVH ≥ 50 mL, *p* = 0.005), the total amount of intraventricular and intraparenchymal hemorrhage (IVH + ICH ≥ 90 mL, *p* = 0.019), the IVH Ratio (IVH Ratio ≥ 40%, *p* = 0.003), posterior circulation aneurysms (*p* = 0.019), presence of spot sign on initial CT scan angiography (*p* = 0.015).

Nonetheless, when the multivariable analysis was performed, only IVH Ratio (*p* = 0.005; OR 3.97), posterior circulation aneurysms (*p* = 0.008; OR 4.05) and spot sign (*p* = 0.022; OR 6.87) turned out to be independent predictors of 30-day mortality.

**Conclusion:**

The risk of mortality in poor-grade aSAH remains considerable despite maximal treatment. Notwithstanding the limitations of a retrospective study, our report highlights some neuroradiological features that in the emergency setting, combined with leading clinical and anamnestic parameters, may support the multidisciplinary team in the difficult decision-making process and communication with family members from the earliest stages of poor-grade aSAH. Further prospective studies are warranted.

## Introduction

Aneurismal subarachnoid hemorrhage (aSAH) remains a detrimental condition despite advances in treatment. Patients with poor clinical status (World Federation of Neurosurgical Societies WFNS IV and V) represent approximately 20 and 40% (1) of hospitalized aSAH patients, respectively, and have a significant overall mortality rate, ranging from 26 to 43% (2), and an unfavorable clinical outcome. For these reasons, patients with poor-grade aSAH have often been considered for withdrawal of life-saving equipment based on the prognostication from the initial neurological assessment. Over the past 20 years, exceptional advances in diagnostic imaging (computed tomography CT, CT angiography, Digital Subtraction Angiography DSA) and treatment (surgical, endovascular and neurocritical care) have not yet shifted the paradigm toward significantly better clinical outcomes.

In this scenario, we would need simple parameters to support the decision-making process and identify patients who harbor a higher probability of survival from this acute event.

The hemorrhagic burden, with its subarachnoid, intraventricular and intraparenchymal counterparts, has been associated with the development of delayed cerebral ischemia (3, 4) and the severity of subarachnoid hemorrhage (5, 6). For these reasons, among many others, radiological parameters, especially in the early phases of the acute event, could be investigated as outcome prognosticators.

The aim of the present study is to investigate the baseline risk factors (clinical, radiological and laboratory) as independent outcome predictors of 30-day mortality in patients with poor-grade aSAH.

## Materials and methods

### Study design and setting

We retrospectively reviewed the clinical, treatment (surgical and endovascular) and outcome (30-day mortality) data of patients with poor-grade aSAH treated at our institution (IRCCS Istituto delle Scienze Neurologiche di Bologna, Bologna, Italy) from December 2010 to December 2020.

### Participants and study size

All patients treated in the early phase were retrospectively reviewed. The eligibility criteria included (a) aSAH with WFNS IV or V on admission after stabilization of vital parameters (7), (b) age of ≥18 years, and (c) treatment within 24 h of hospital admission. Patients with (a) out-of-hospital cardiac arrest (8, 9), (b) traumatic, mycotic or arteriovenous malformation-related aneurysms, (c) clinical signs of severe brainstem dysfunction (bilaterally dilated pupils, no corneal reflexes), (d) pregnancy, were excluded from this analysis.

For all patients, family members signed an informed consent for the scientific use of the data according to the requirements of the local Institutional Review Board.

### Data sources

Clinical and treatment data were collected in a digital archive. Follow-up information was obtained by trained personnel, blinded to the results of the present study during outpatient clinical evaluations or telephone interviews with patients, their relatives, or their general practitioners at 1 month after surgery.

### Treatment protocol

The treatment protocol has been described in part elsewhere (10). As per our Institutional protocol we offer treatment to all poor-grade aSAH patients in the early (24 h) phase after hospital admission, except some with clinical signs of severe brainstem dysfunction. This strategy is conducted for the purpose of preventing aneurysm rebleeding. The neurocritical and early treatment strategy was in agreement with the international aSAH guidelines published at the time of the last patient recruitment (11, 12).

Briefly, all patients included in this study were centralized at our Institution after early stabilization of vital parameters. If acute hydrocephalus was present on the initial CT scan, an external ventricular drainage (EVD) was placed prior to aneurysm treatment. All patients underwent early CT angiography and multidisciplinary discussion (with at least one senior Interventional Neuroradiologist, one senior Vascular Neurosurgeon and one senior Neuroanesthesiologist with experience in aSAH management) of the indication and type of treatment based on clinical condition, comorbidities, and angioarchitectural features of the aneurysm. For patients whose aneurysms were amenable to either endovascular or surgical treatment, coiling was identified as the preferred treatment modality. Diagnostic DSA was performed in patients who did not reveal an aneurysm on CT angiography, or to identify and characterize perforating arterial branches from the aneurysmal neck before surgery, or, in any case, before planned endovascular treatment as part of the procedure.

Neurocritical care, prevention and treatment of vasospasm and delayed cerebral ischemia (DCI) were provided to all patients included in the study according to guidelines (11, 12).

The effectiveness of aneurysm treatment was investigated by postoperative CT angiography or DSA (in selected challenging cases) for surgically treated patients and with magnetic resonance angiography (MRA) for those patients who received endovascular treatment.

Patients who developed symptomatic radiologic hydrocephalus, in the subacute phase after EVD removal, underwent ventriculo-peritoneal (VP) shunt placement.

### Assessment quantitative variables and outcome

We collected demographic data (age, sex), clinical and laboratory data before treatment (including on admission WFNS grade stabilization of vital parameters, mean arterial blood pressure MAP, presence of seizure at symptom onset, serum glucose level, PaO_2_/FiO_2_ Ratio for respiratory failure, presence of antiplatelet/anticoagulant use, presence of pupillary light reflex PLR, white blood cell count WGC), radiological parameters on initial CT scan and CT angiography, CTA (modified Fisher scale mFS, number and type of ventricles filled with blood, presence of intraventricular hemorrhage IVH, IVH volume, presence of intraparenchymal hemorrhage ICH, ICH volume, total amount of ICH + IVH, IVH ratio calculated as [IVH/ (total amount of ICH + IVH)], presence of acute hydrocephalus, presence of midline shift, presence of subdural or extradural hematoma, location of aneurysm, presence of spot sign on CTA, any involvement of the parental arteries in the aneurysmal neck), treatment-related parameters (time from symptom onset to treatment, type of treatment, placement of external ventricular drainage EVD), presence of rebleeding at any time (before or after treatment), complications (delayed cerebral ischemia (13), hydrocephalus requiring permanent shunt placement).

Detailed volumetric assessment of intracranial hemorrhage, semi-automated segmentation and volumetric assessment were performed on non-contrast enhanced brain CT scans by two different neuro-radiologists blinded to patient characteristics and outcome. The neuroradiological analysis was carried out using the Advantage Workstation (AW) Server 3.2 (GE Healthcare, Chicago, Illinois, United States). Bleeding volumes were calculated in milliliters for the ICH and the IVH counterparts by multiplying the slice thickness by the hemorrhagic region. The IVH ratio was calculated as [IVH /total amount of ICH + IVH] and expressed as a percentage. Clinical outcome was defined as mortality at 1 month.

### Statistical analysis

For all pre-treatment (demographic, clinical and laboratory data), radiological, and treatment-related parameters, rebleeding and complications, percentages of frequency distributions were analyzed. For statistical analysis, variables were sorted as indicated below. Variables were transformed into binary variables to be used in the univariable and multivariable analyses. The dichotomous variables were sex, WFNS grade, seizure, antiplatelet/anticoagulant, presence of PLR, presence of IVH, presence of ICH, acute hydrocephalus, midline shift, subdural/extradural hematoma, spot sign, involvement of the parental arteries in the aneurysm neck, type of treatment, EVD, rebleeding, delayed cerebral ischemia, and shunt placement. For non-dichotomous variables, cut-off values were chosen according to clinical/radiological criteria and published data. Subsets for predictors and outcome variables were as follows: age, < 65 years Vs. ≥ 65 years; MAP, < 90 mmHg Vs. ≥ 90 mmHg; serum glucose level, <180 mg/dL Vs. ≥ 180 mg/dL; PaO_2_/FiO_2_ Ratio, < 200 Vs. ≥ 200; WGC, < 15 × 10^9^ ≥ 15 × 10^9^/L; mFS, 3 Vs. 4; number of ventricles filled with blood, < 3 Vs. ≥ 3; IVH volume, < 50 cc Vs. ≥ 50 mL; ICH volume, < 30 cc Vs. ≥ 30 mL; ICH + IVH volume, < 90 cc Vs. ≥ 90 mL; IVH ratio, < 40% Vs. ≥ 40%; aneurysm location, anterior circulation Vs. posterior circulation; time from symptom onset to treatment, < 6 h Vs. ≥ 6 h.

For univariable analysis, contingency tables and Pearson’s Chi-squared test were used to evaluate the association between the categorical variables and the outcome. Results were presented as absolute (n) and relative frequencies (%). Multivariable analysis was performed using the step-wise logistic regression model, including only variables that were statistically significant in the univariable analysis. Results were presented as Odds Ratio (OR) and 95% Confidence Interval (95% CI). Statistical significance was defined as a *p* value <0.05. Statistical analysis was performed using Stata 14.2.

## Results

### Participants and descriptive data

A total of 149 patients with poor-grade aSAH were included in this study. None of the patients in this series presented with an in-hospital cardiac arrest. The mean age was 61.3 (SD 11.9 years). In total, 47 patients (32%) presented with WFNS grade 4 and 102 patients (68%) with WFNS grade V. A total of 126 patients (85%) harbored an aneurysm located in the anterior circulation and 23 patients (15%) in the posterior circulation. A total of 99 patients (66%) were treated surgically while endovascular treatment was performed in 50 cases (44%). The overall rebleeding rate (at any time, before and after treatment) was 11%.

Tables 1, 2 summarize the clinical/laboratory variables on admission and the radiological parameters analyzed.

**Table 1:** Clinical and laboratory features on admission.

Clinical and laboratory variables	No. of patients (%)	Survivors	Non-survivors
Sex			
Male subjects	41 (28%)	33 (30%)	8 (26%)
Female subjects	108 (72%)	85 (70%)	23 (74%)
WFNS score			
IV	47 (32%)	39 (33%)	8 (26%)
V	102 (68%)	79 (67%)	23 (74%)
Seizure presentation			
No	138 (93%)	107 (91%)	31 (100%)
Yes	11 (7%)	11 (9%)	0
Onset of neurological deficits			
No	88 (59%)	66 (56%)	22 (71%)
Yes	61 (41%)	52 (44%)	9 (29%)
Pupillary reflex			
Normal	117 (79%)	95 (81%)	22 (71%)
Abnormal	32 (21%)	23 (19%)	9 (29%)
Mean Arterial Blood Pressure (MAP)			
< 90 mmHg	116 (78%)	90 (76%)	26 (84%)
≥ 90 mmHg	33 (22%)	28 (24%)	5 (16%)
Glycemia			
< 180 mg/dL	90 (60%)	77 (65%)	13 (42%)
≥ 180 mg/dL	59 (40%)	41 (35%)	18 (58%)
White Blood Cell (WBC) count			
< 15 × 10^9^/L	93 (62%)	79 (67%)	14 (45%)
≥ 15 × 10^9^/L	56 (38%)	39 (33%)	17 (55%)
PaO_2_/FiO_2_ (P/F) ratio			
≥ 200	117 (79%)	94 (80%)	23 (74%)
< 200	32 (21%)	24 (20%)	8 (26%)
Anticoagulant therapy			
No	146 (98%)	116 (98%)	30 (97%)
Yes	3 (2%)	2 (2%)	1 (3%)
Antiplatelet therapy			
No	121 (81%)	97 (82%)	24 (77%)
Yes	28 (19%)	21 (18%)	7 (23%)

WFNS, World Federation of Neurosurgical Societies.

**Table 2:** Radiological parameters.

Radiological variables	Total (149 patients)	Survivors (118 patients)	Non-survivors (31 patients)
mFISHER scale			
3	23 (15%)	21 (18%)	2 (6%)
4	126 (85%)	97 (82%)	29 (94%)
IVH volume			
< 50 mL	132 (89%)	109 (92%)	23 (74%)
≥ 50 mL	17 (11%)	9 (8%)	8 (26%)
ICH volume			
< 30 mL	112 (75%)	86 (73%)	26 (84%)
≥ 30 mL	37 (25%)	32 (27%)	5 (16%)
ICH + IVH total volume			
< 90 mL	139 (93%)	113 (96%)	26 (84%)
≥ 90 mL	10 (7%)	5 (4%)	5 (16%)
IVH Ratio			
< 40%	69 (46%)	62 (53%)	7 (23%)
≥ 40%	80 (54%)	56 (47%)	24 (77%)
Number of ventricles filled with blood			
< 3	46 (31%)	39 (33%)	7 (23%)
≥ 3	103 (69%)	79 (67%)	24 (77%)
Type of IVH			
Monoventricular	10 (8%)	8 (8%)	2 (7%)
Biventricular	12 (10%)	12 (12%)	0
Triventricular	11 (9%)	7 (7%)	3 (10%)
Tetraventricular	33 (26%)	26 (26%)	7 (24%)
Acute hydrocephalus			
No	38 (26%)	29 (25%)	9 (29%)
Yes	111 (74%)	89 (75%)	22 (71%)
Midline shift			
< 5 mm	98 (66%)	81 (69%)	17 (55%)
≥ 5 mm	51 (34%)	37 (31%)	14 (45%)
Subdural / extradural hematoma			
No	120 (81%)	93 (79%)	27 (87%)
Yes	29 (19%)	25 (21%)	4 (13%)
Aneurysm location			
Anterior circulation	126 (85%)	104 (88%)	22 (71%)
Posterior circulation	23 (15%)	14 (12%)	9 (29%)
Spot sign			
No	142 (95%)	115 (97%)	27 (87%)
Yes	7 (5%)	3 (3%)	4 (13%)
Vessel involvement in aneurysmal neck			
No	114 (77%)	90 (76%)	24 (77%)
Yes	35 (23%)	28 (24%)	7 (23%)

IVH, Intraventricular Hemorrhage. ICH, Intraparenchymal Hemorrhage. IVH Ratio, [IVH / (IVH + ICH)].

A total of 126 patients (85%) presented with IVH on the initial CT scan and 111 patients (74%) presented with acute hydrocephalus requiring emergent EVD placement. In total, 107 patients (72%) had some degree of ICH on the admission CT scan. Among radiological variables, the mean volumes of IVH, ICH and the total amount of IVH + ICH were 17.5 (SD 24.7) mL, 18.6 (SD 23.6) mL, and 36.1 (SD 32.6) mL, respectively. The mean IVH ratio was 49.4 (SD 40.6) %.

### Main results, outcome data and predictors of 30-day mortality

The overall mortality rate at 1 month after poor-grade aSAH was 21%. The causes of mortality were related to the aSAH or complications related to the aSAH in all cases. In total, 59 patients (40%) presented with delayed cerebral ischemia and 51 patients (34%) developed hydrocephalus during the follow-up period (1 month), requiring ventriculo-peritoneal placement. Of the 32 patients (21% of the total) who presented with pupillary reflex abnormalities on admission, 9 patients (6%) did not resolve after aneurysm treatment and they were non-survivors at 1 month after aSAH.

**Figure 1:**
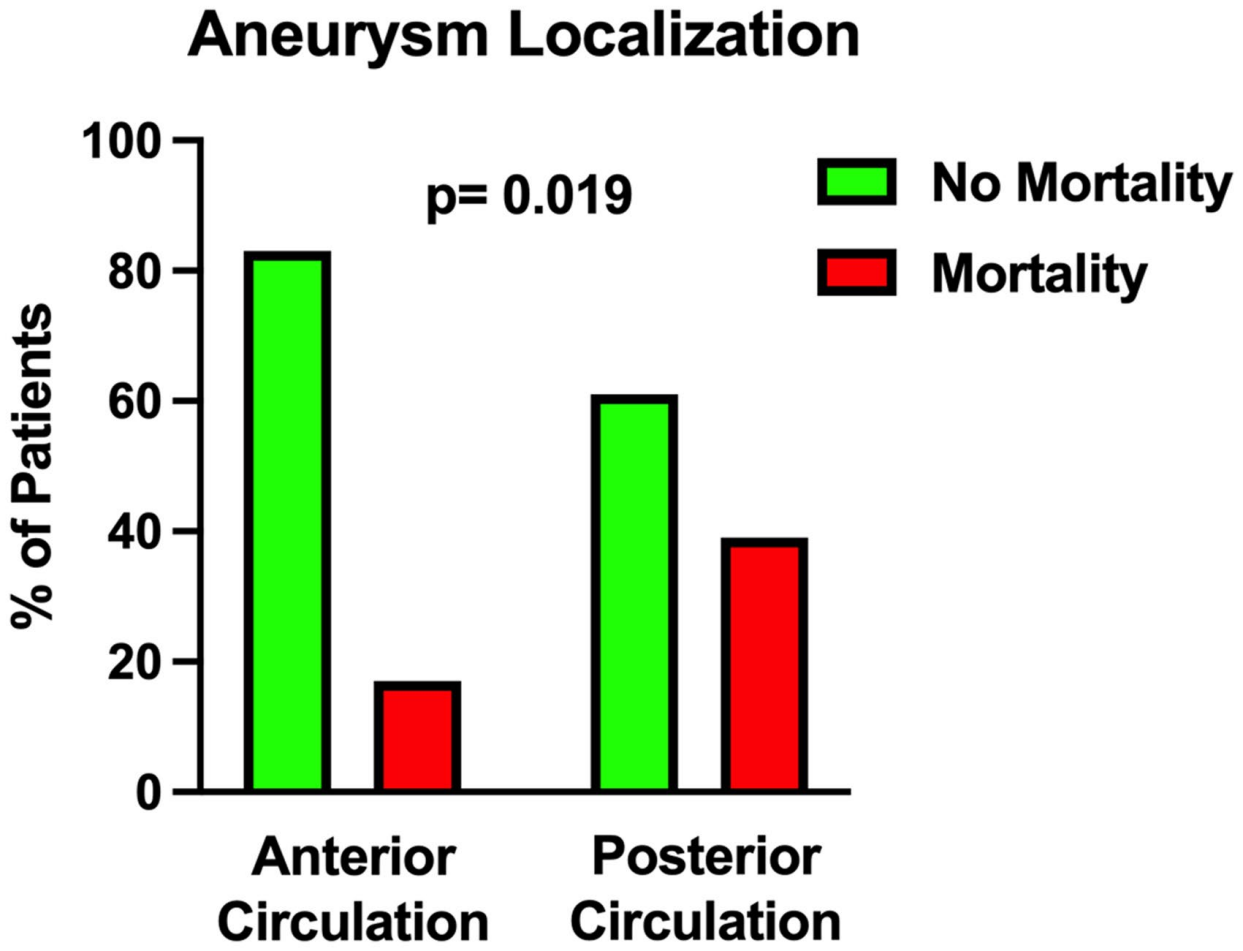
Univariable analysis showing the correlation between posterior circulation aneurysm location and mortality.

**Figure 2:**
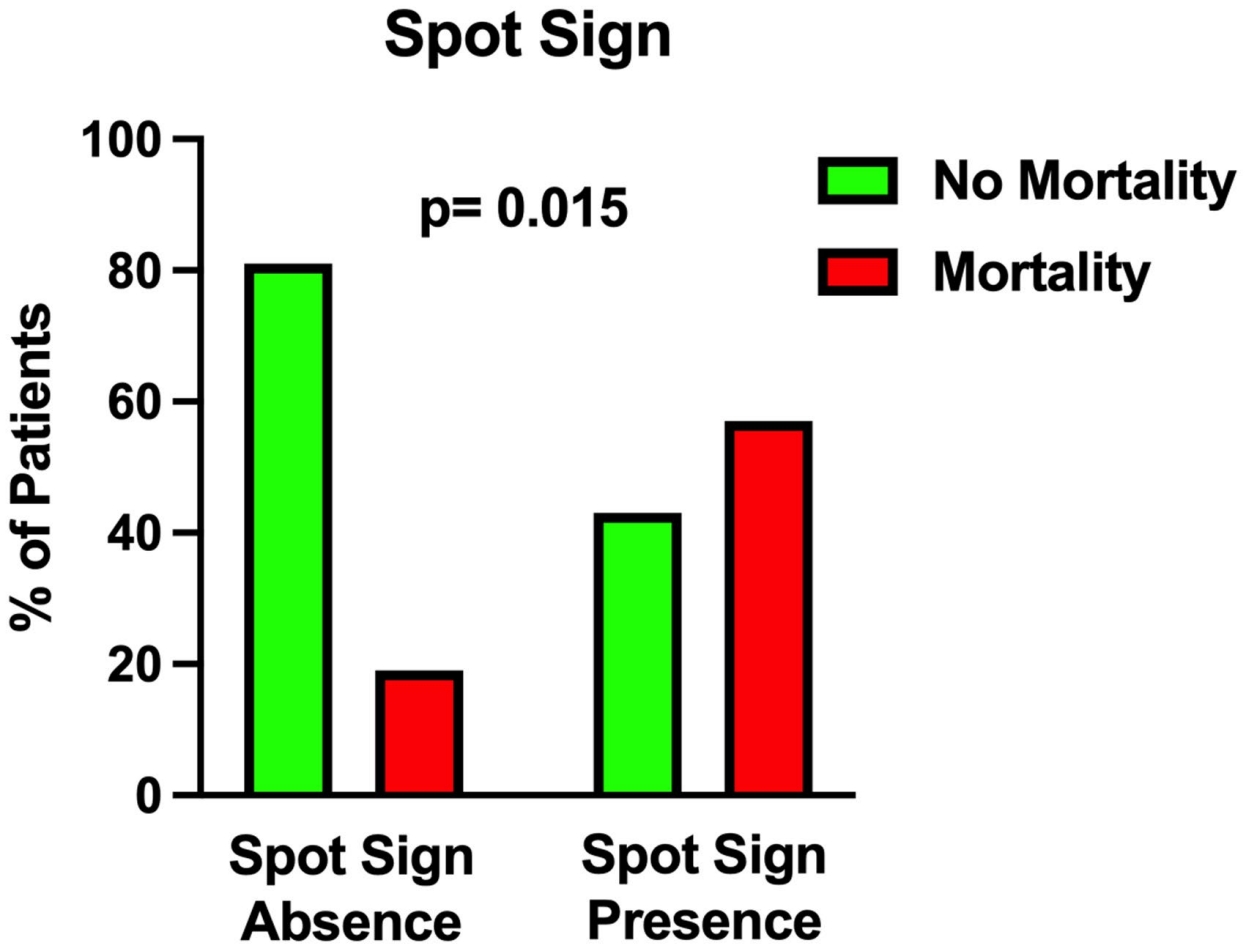
Univariable analysis showing the correlation between spot sign presence and mortality.

**Figure 3:**
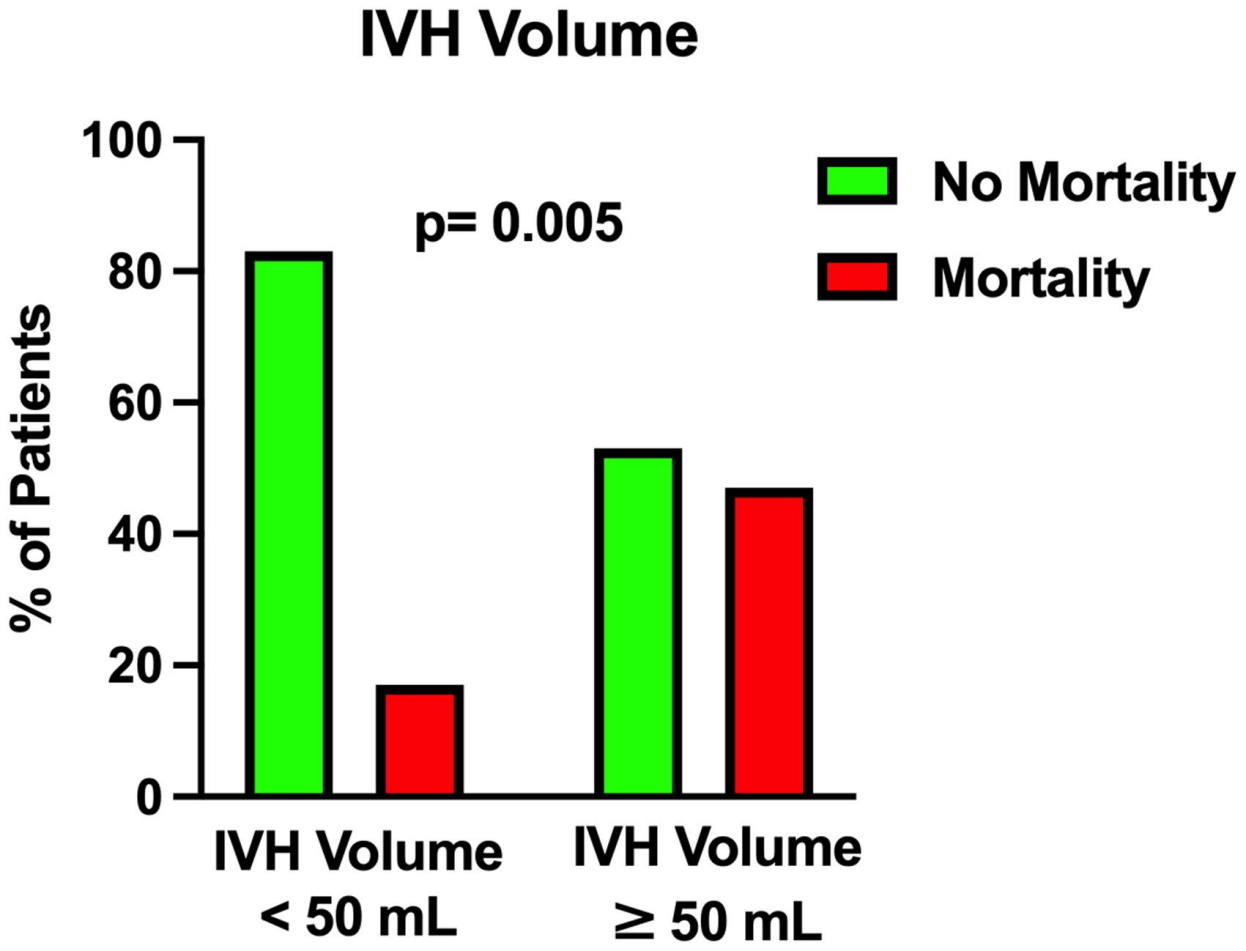
Univariable analysis showing the correlation between intraventricular hemorrhage volume and mortality.

**Figure 4:**
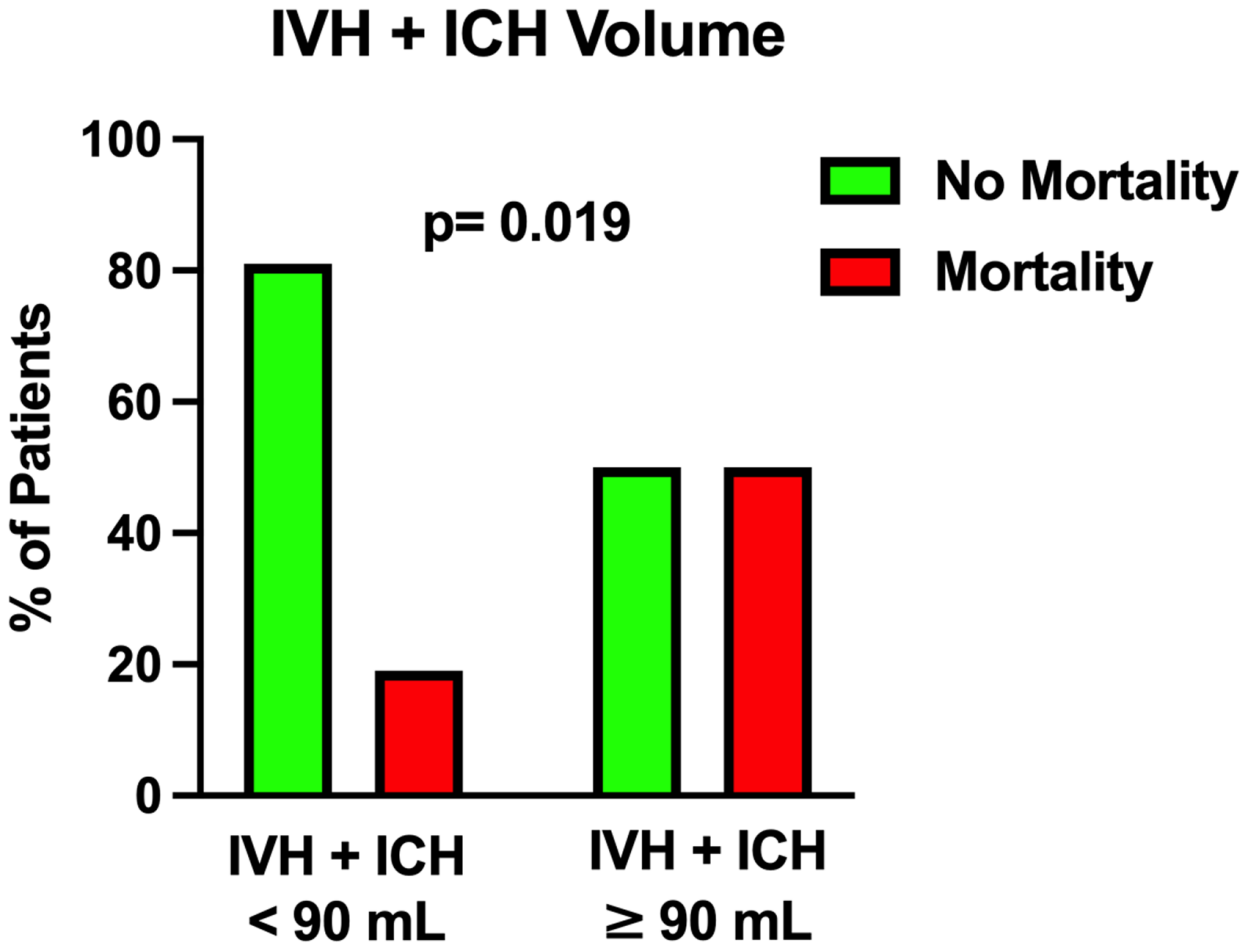
Univariable analysis showing the correlation between the total amount of intraventricular hemorrhage and intraparenchymal hemorrhage volume and mortality.

**Figure 5:**
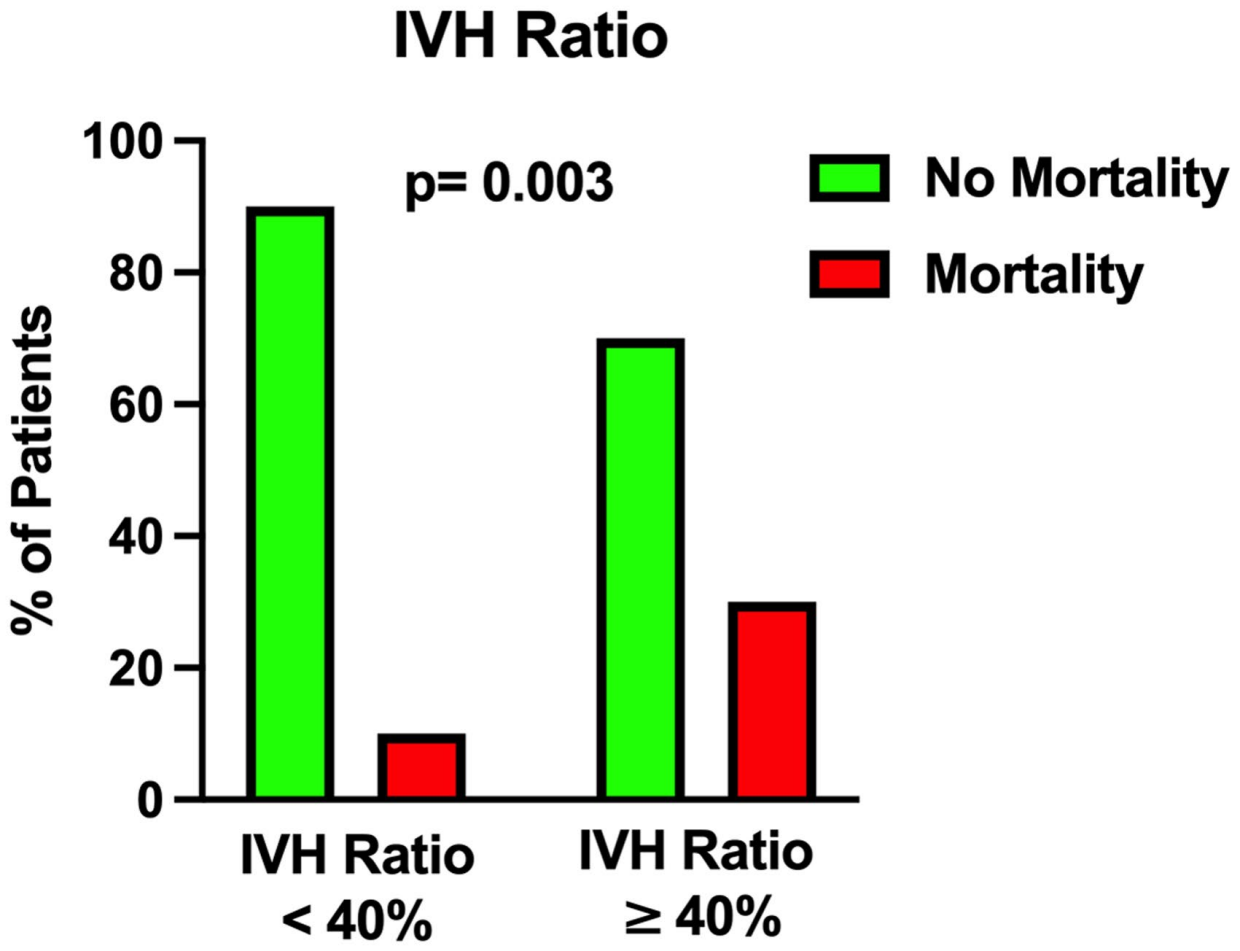
Univariable analysis showing the correlation between intraventricular hemorrhage ratio (see the text) and mortality.

The univariable statistical analysis identified five variables associated with the likelihood of death. The mortality rate was higher in patients harboring a posterior circulation aneurysm in comparison with patients with anterior circulation aneurysms (39% Vs. 17%, *p* = 0.019). On the initial CTA a spot sign emerged in 7 patients (5%), of whom 57% were dead at 1 month and 43% were alive (*p* = 0.015). Investigating the quantitative radiological analysis, IVH volume ≥ 50 mL (*p* = 0.005), IVH + ICH volume ≥ 90 mL (*p* = 0.019), and IVH Ratio ≥ 40% (*p* = 0.003) were revealed to be associated with mortality at 1 month. Figures 1–5 depict the results of the univariable analysis for variables that were found to be significant.

**Figure 6:**
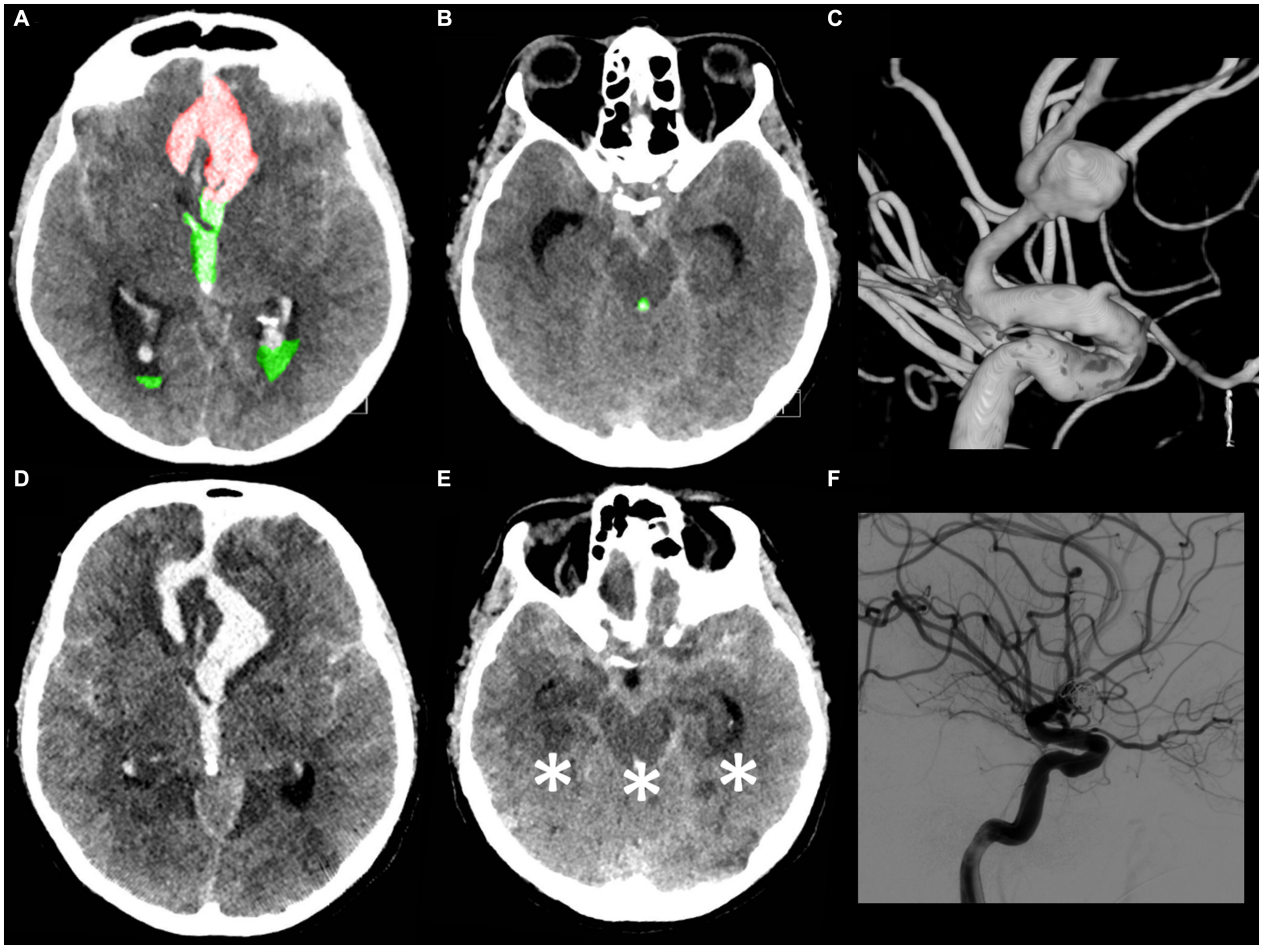
A 53-year-old male patient presented with a loss of consciousness. The WFNS score evaluation on admission was V with isochoric pupils and an intact pupillary light reflex. The initial CT scan **(A,B)** demonstrated the presence of a mFisher grade 4 aSAH with intraventricular hemorrhage (green **A,B**), volume 30 mL, and acute hydrocephalus with intraparenchymal hemorrhage (red **A**), volume 7 mL (IVH ratio 81%). The CT angiography and digital subtraction angiography **(C)** showed a ruptured communicating artery aneurysm. An urgent external ventricular drainage was placed and the aneurysm was endovascularly treated within 5 h of symptom onset. The 72 h post-treatment CT scan (**D,E** in corresponding slices as compared, respectively, with **A,B**) revealed the presence of bilateral temporal ischemia, occipital ischemia, and pontomesencephalic ischemia (asterisk **E**). **(F)** Post-coiling digital subtraction angiography showing aneurysm occlusion. The patient died 5 days after aSAH.

Nonetheless, when multivariable analysis was performed, only IVH Ratio ≥ 40% (*p* = 0.005; OR 3.97; 95% CI 1.52–10.36), posterior circulation aneurysms (*p* = 0.008; OR 4.05; 95% CI 1.44–11.37) and spot sign presence (*p* = 0.022; OR 6.87; 95% CI 1.32–35.88) turned out to be independent predictors of 30-day mortality (Table 3). A case example is illustrated in Figure 6.

**Table 3:** Multivariable logistic model using 30-day mortality as the outcome.

30-day mortality	Odds ratio	*p*	95% confidence interval
IVH ratio ≥ 40% vs. < 40%	3.97	0.005	1.52–10.36
Posterior circulation vs. anterior circulation aneurysm	4.05	0.008	1.44–11.37
Spot sign presence vs. absence	6.87	0.022	1.32–35.88

IVH, Intraventricular Hemorrhage.

## Discussion

### Key results

In this single-center retrospective study, we investigated the predictors of 30-day mortality in a series of patients with poor-grade aSAH treated surgically or endovascularly over one decade. Our analysis revealed that the ratio of IVH volume to the total amount of IVH and ICH counterparts (“IVH ratio”), the presence of a spot sign on the initial CTA and the localization of the aneurysm in the posterior circulation were strongly associated with the occurrence of mortality within 1 month after the acute event.

### Interpretation

In the field of cerebrovascular disease, poor-grade aSAH accounts for a large percentage of neurological disability and, consequently, a high burden on the community and the patient’s caregivers (2, 14, 15).

In the literature, different reports and scores (16, 17) have addressed the factors influencing good and poor outcomes in aSAH and, in recent years, some studies have specifically considered the subset of poor-grade aSAH patients.

Notably, in 2022 de Winkel et al. published a systematic review and meta-analysis (18) on early predictors of functional outcome in poor-grade aSAH. This analysis revealed that WFNS grade or Hunt and Hess (HH) grade IV, presence of clinical improvement before aneurysm treatment and intact pupillary light reflex were associated with the likelihood of favorable outcome, while, older age, increasing modified Fisher grade and the presence of intracerebral hematoma on admission imaging decreased the possibility of favorable outcome (18). Moreover, in 2021 Shen et al. (2) identified a scoring model to predict functional outcomes in poor-grade aSAH including different significant predictors (modified Fisher grade > 2, age ≥ 65 years, conservative treatment, WFNS grade V, delayed cerebral ischemia, shunt-dependent hydrocephalus and cerebral herniation) for poor prognosis (modified Rankin Scale ≥3). According to this score model, patients were divided into three categories: low risk (0–1 points), intermediate risk (2–3 points), and high risk (4–9 points), with predicted risks of poor prognosis of 11, 52, and 87%, respectively (2).

As a matter of fact, notwithstanding the advancements in techniques and management that ideally allow for intensive and adequate treatment, the mortality and morbidity rates in patients with poor-grade aSAH remain very high. The decision-making process during the early phases of an acute cerebrovascular event is arduous and the proper indication to treat and resource distribution results are very challenging.

Few studies have focused on the factors affecting mortality in poor-grade aSAH. Table 4 summarizes the results of the most relevant reports highlighting this specific focus (1, 5, 15, 19–24).

**Table 4:** Literature review of mortality predictors in patients with poor-grade aneurysmal subarachnoid hemorrhage.

First author and year	No. pts	Population	Treatment (%)	IVH (%)	ICH (%)	Mortality rate	Factors affecting mortality
Yoshimoto et al., 1997	68	WFNS IV (56%) or WFNS V (44%)	No treatment (40%) NCH (60%)	NA§	NA§	50% at discharge	Unreactive pupils on admission No motor response on admission
Szklener et al., 2015	101	WFNS IV (26%) or WFNS V (74%)	No treatment (100%)	63%	NA§	73% at 1 month	WFNS grade, Age, Fisher grade, Leukocytosis
van Lieshout et al., 2016	61	WFNS V	NA§	NA§	NA§	28% in-hospital mortality	Delayed transport to specialized neurosurgical care Cardiovascular complications
Cai et al., 2017	58	WFNS IV (70,2%) or WFNS V (29,8%)	NRD (100%)	46%	NA§	27% at 6 months	Hunt-Hess grade, Age, Fisher grade, Systolic blood pressure variability-successive variation
Panni et al., 2019	63	WFNS IV (44,4%) or WFNS V (55,6%)	NCH (31,7%) NRD (68,3%)	82,5%	NA§	30,2% at 12 months	Higher volume of global intracranial bleeding (cutoff 51 mL), the presence of global cerebral edema on admission CT scan
Xie et al., 2019	66	HH IV (58%) or HH V (42%)	NCH (100%)	74%	46%	27% at 6 months	HH grade V (*p* = 0,04) Admission serum fibrinogen level (*p* = 0,006)
Lashkaret al., 2020	176	HH V	No treatment (36%) NCH (32,9%) NRD (31,1%)	79,1%	52,5%	65,8% at 12 months	Older age No treatment
Gouvêa Bogossian et al., 2021	353	WFNS IV (32%) or WFNS V (68%)	NA§	94%	NA§	57% in-hospital mortality	Age, SOFA Score, WFNS 5, Endovascular treatment, Prophylactic nimodipine, Intracranial hypertension, Hydrocephalus
Duan et al., 2022	116	WFNS IV (35%) or WFNS V (65%)	NCH (100%)	96,3%	NA§	35% in-hospital mortality	Age ≥ 65 years, pupillary changes, delayed cerebral ischemia, use of Subarachnoid Hemorrhage Early Brain Edema Score for management of decompressive craniectomy
Present study	149	WFNS IV (32%) or WFNS V (68%)	NCH (66%) NRD (44%)	85%	72%	21% at 1 month	IVH Ratio ≥ 40% (*p* < 0,005) Posterior circulation aneurysms (*p* < 0,008) Spot Sign presence (*p* < 0,022)

pts, patients; IVH, Intraventricular Hemorrhage; ICH, Intraparenchymal Hemorrhage; SAH, Subarachnoid Hemorrhage; WFNS, World Federation of Neurosurgical Societies; HH grade, Hunt-Hess grade; NCH, Neurosurgical Treatment; NRD, Neuroradiological Treatment; SOFA, Sequential Organ Failure Assessment; NA: Not Available.

In this scenario, our study identified some radiological parameters that may assist the intervening interdisciplinary team in planning ultra-early management of patients with poor-grade aSAH..

Among the parameters found to be significantly correlated with 30-day mortality, the most interesting is the IVH ratio, which is the ratio of IVH volume to the total amount of IVH and ICH counterparts. In 2019, Panni et al. performed a detailed volumetric analysis of different bleeding distributions in poor-grade aSAH (5). These authors analyzed intracranial bleeding with its subarachnoid, intracerebral and intraventricular portions. They showed that: (1) global intracranial bleeding (sum of the three components) emerged as an independent predictor of good outcome (cutoff 24 mL); (2) relative percentage of ICH in global volume (10% of total) and pure SAH (64% of total) emerged as independent predictors of worse and better outcome, respectively; (3) global bleeding volume (cutoff 51 mL) along with global cerebral edema showed to independently predict 12-month mortality (5).

Several studies in the literature support the fact that IVH (25) and ICH (16, 18, 26) are negative and critical prognostic factors in aSAH. Compared to the subarachnoid counterpart, we found out that routine analysis of IVH and ICH fractions is a simple, fast and relatively easy-to-learn method for quantitative assessment of the hemorrhagic load on the initial CT scan. In particular we found that an IVH ratio ≥ 40% is an independent predictor of mortality at 1 month after poor-grade aSAH.

These considerations can be implemented in the emergency scenario of poor-grade aSAH patients and, when combined with leading clinical and anamnestic information, can support the multidisciplinary neurovascular team in formulating clinical decisions and in a more informed discussion with the families during these delicate and eventful stages.

A further parameter that has been associated with mortality is the location of the aneurysm in the posterior circulation. The poor prognosis of ruptured intracranial aneurysms of the posterior circulation has been highlighted in previous studies (27, 28). In 2012 Inamasu et al. published a report (29) about acute cardiopulmonary dysfunction, particularly neurogenic pulmonary edema (NPE) and takotsubo-like cardiomyopathy (TLC) in poor-grade aSAH. They discovered that ruptured posterior circulation aneurysms were predictors of NPE and TLC (29) which may lead to an inauspicious outcome. Another reason may be related to the more frequent localization of dissecting aneurysms in the posterior circulation and their correlation with aneurysmal rebleeding that may lead to a fatal outcome (30, 31).

Finally, the presence of a spot sign on the initial CTA was found to be related to 1-month mortality. In 2017 Burkhardt et al. investigated the role of CTA spot signs in aSAH patients (32). In their multicenter study, patients with spot sign-positive aneurysmal ICH exhibited larger ICH volumes and a higher rate of intraprocedural aneurysm rupture; nevertheless, the long-term outcome was comparable to that of spot sign-negative ICH patients (32). As highlighted in that study (32), there are different theories about the pathophysiology of spot signs in aSAH. Some authors speculate that it is an indirect sign of vascular fragility affecting the small vessels around the ICH, including the aneurysm; other authors support the hypothesis that the dynamic of lightning-quick bleeding increases the possibility of “shear stress” damaging the surrounding ICH tissue and, as a consequence, the small vessels around the ICH (32).

### Limitations

As far as limitations are concerned, the retrospective and single-center nature of the present study inevitably introduces selection and expertise biases that are naturally related to these study designs. Furthermore, we acknowledge that a time frame of 10 years, a relatively limited number of patients, the absence of long-term outcomes and the lack of data on functional outcomes, may decrease the strength of our findings.

## Conclusion

The risk of death in poor-grade aSAH remains considerable despite maximal treatment. Notwithstanding the limitations of a retrospective study, our report points out some neuroradiological features that in the emergency setting, combined with leading clinical and anamnestic parameters, may support the multidisciplinary team in the difficult decision-making process and communication with family members from the earliest stages of poor-grade aSAH. Further prospective studies are warranted.

## Data availability statement

The raw data supporting the conclusions of this article will be made available by the authors, without undue reservation.

## Ethics statement

The studies involving humans were approved by IRCCS Istituto delle Scienze Neurologiche di Bologna. The studies were conducted in accordance with the local legislation and institutional requirements. Written informed consent to participate in this study was provided by the next of kin in accordance with the requirements of the Ethics Board of IRCCS Istituto delle Scienze Neurologiche di Bologna.

## Author contributions

AS: Data curation, Writing – original draft, Writing – review & editing. AR: Data curation, Writing – original draft, Writing – review & editing. ML: Data curation, Writing – review & editing. CZ: Data curation, Formal analysis, Methodology, Writing – review & editing. LB: Data curation, Formal analysis, Methodology, Writing – review & editing. MD: Writing – review & editing. CP: Writing – review & editing. ANC: Writing – review & editing. RA: Writing – review & editing. EM: Writing – review & editing. MM: Writing – review & editing. CS: Writing – review & editing. CC: Writing – review & editing. ALC: Writing – review & editing. CB: Writing – review & editing. LC: Writing – review & editing.
